# The association between renal function and structural parameters: a pig study

**DOI:** 10.1186/1471-2369-9-18

**Published:** 2008-12-23

**Authors:** Anders B Lødrup, Kristian Karstoft, Thomas H Dissing, Jens R Nyengaard, Michael Pedersen

**Affiliations:** 1 MR Research Center, Aarhus University, Aarhus, Denmark; 2 Stereology and Electron Microscopy Research Laboratory and MIND Centre, Aarhus University, Aarhus, Denmark; 3 Institute of Clinical Medicine, Aarhus University, Aarhus, Denmark

## Abstract

**Background:**

The objective was to investigate the association between renal structural parameters and renal function. The structural parameters were renal cortical volume, total renal volume, number of glomeruli, and total glomerular volume, and renal function was expressed by the single kidney GFR (skGFR). Investigations were performed using both healthy and chronically diseased kidneys. We investigated which of the structural parameters showed the best correlation to renal function and evaluated the possibility of predicting the renal function from structural parameters.

**Methods:**

Twenty-four pigs, twelve with healthy kidneys and twelve with diseased kidneys, underwent skGFR measurements. Nephrectomies were performed and structural parameters were estimated using stereological procedures. The correlation between the structural parameters and skGFR was analysed by Pearson's correlation test. The prediction of skGFR from structural parameters was analysed by a linear regression test.

**Results:**

In general, we demonstrated a good correlation between structural parameters and skGFR. When all kidneys were evaluated together Pearson's correlation coefficient between skGFR and any stereological parameter was above 0.60 and highly significant (p < 0.001), and with r-values ranging from 0.62 regarding number of glomeruli, to 0.78 regarding cortical volume. The best correlation was found between cortical volume and skGFR. Prediction of single kidney GFR from any structural parameter showed to be quite imprecise.

**Conclusion:**

The observed correlations between structural parameters and renal function suggest that these parameters may potentially be useful as surrogate markers of the renal function. At present, however, precise prediction of renal function based on a single structural parameter seems hard to obtain.

## Background

The pathology of chronic kidney disease usually involves structural and functional changes, often interconnected, and according to the hyperfiltration theory, as proposed by Brenner et al, a reduction in the number of glomeruli (N(glom)) leads to hemodynamic changes associated with glomerular hypertension, hyperfiltration and proteinuria [1].

The relationship between renal structural and functional parameters is still not fully understood. Widjaja et al concluded that three-dimensional structural parameters such as total kidney volume (V(total)) correlates better to the kidney function than two-dimensional structural parameters [2]. Volume of cortex (V(cor)) has been shown to diminish over time in patients with chronically injured kidneys [3]. N(glom) has been shown to determine the long time allo-graft outcome [4] and the renal filtration capacity in diabetic patients has been shown to correlate to the total surface area of the glomerular capillaries in the kidney [5]. In a study involving renal allograft biopsies from lean and obese donors, the latter presented a greater glomerular volume (V(glom)) and an increased GFR, suggesting a correlation between the two parameters [6]. In patients with insulin-dependent diabetes mellitus (IDDM) and albuminuria, V(glom) was significantly increased, something not seen in IDDM patients without albuminuria [7]. In accordance with this, a negative correlation has been demonstrated between N(glom) and severe diabetic glomerulonephropathy [8].

Hence, different studies have described the relationship between renal structural parameters and kidney function, but to our knowledge, no studies have evaluated which of the above mentioned structural parameters shows the best correlation to kidney function.

In this study the objective was to investigate the correlation between the quantitative parameters V(cor), V(total), N(glom), and V(glom) and the renal function for healthy as well as chronically diseased pig kidneys, and, in relation to this, determine which of the structural parameters showed the best correlation to renal function. Secondly, the objective was to evaluate the possibility of predicting skGFR from structural parameters.

Our choice of methods was determined by the need for reproducible, valid and unbiased results. Total unilateral ureteral obstruction followed by relief (UUO) was performed in some pigs, and the obstructed kidneys (UUOipsi) as well as the opposite kidneys (UUOcontra) were analysed. In addition, kidneys from healthy pigs were analysed (Controls). Kidney function was estimated as single kidney GFR (skGFR) by direct determination of clearance of ^51^chromium ethylenediaminetetraacetic acid (Cr-EDTA). The structural parameters V(cor), V(total), N(glom) and V(glom) were estimated by the use of design-based stereology (stereological parameters).

## Methods

### Animal handling

Danish Landrace female pigs (n = 24) weighing 29.5 ± 2.3 kg were included in the study. Premedication was at all experimental days given using an im injection of ketamine (10 mg/kg) and midazolam (0.5 mg/kg). The pigs were then intubated with a cuffed endotracheal tube and ventilated using a volume-controlled Servo-ventilator (Siemens, Solna, Sweden) with a gaseous mixture of N_2_0 and 0_2 _(2:1). Anaesthesia was maintained with isoflurane (1.5%) throughout the entire experimental procedure. The animals were allocated into two groups subjected to either UUO (n = 12) or no surgical procedure (Controls) (n = 12).

### Surgical procedures

Pigs subjected to UUO underwent the following experimental procedure: MRI and serum creatinine analysis was performed to ensure that the animal did not present renal abnormalities. The surgical procedures were then advanced by a 4–7 cm subcostal flank incision randomly on the left (n = 6) or right (n = 6) side, the muscle layers were divided, and the ureter was found retroperitoneally. Obstruction was performed using a silicone tube (duodenal feeding tube CH 14, ∅ = 3.0 mm) cut to a length of 3 cm, wrapped around the proximal part of ureter and fixated using ligatures. Seven days later UUO was verified with ultrasonography, showing the presence of hydronephrosis. After that, the obstruction was relieved: The flank incision was opened, the muscle layers were divided, the ligations of the tube were cut through, and the tube was removed. Seven weeks later, each animal underwent skGFR-measurements followed by laparotomic operation including biopsy withdrawel and nephrectomy. Subsequently, the pig was sacrificed. Pigs in the control group underwent investigations equivalent to the procedure for the UUO pigs at the last examination day. The establishment and removal of UUO was performed under sterile conditions.

The study complied with the national regulations for care and use of experimental animals, and the Danish national board regarding animal experiments approved the protocol.

### skGFR measurements

For skGFR measurements, each ureter was catheterised. The ureter was accessed using the same procedure as in the establishment of the ureteral obstruction. The ureter was incised 6–7 cm distally from pelvis, and a silicone tube (feeding tube 10 G, luminal diameter 2.0 mm) was used to catheterize the ureter. A ligature was placed around ureter and the tube in order to obtain patent drainage by the tube. This procedure was performed bilaterally. An arterial entrance was achieved in the profound (deep) femoral artery using an arterial sheath (Radiofocus Introducer II; Terumo, Leuven, Belgium) a.m. Seldinger for blood sampling and monitoring of arterial blood pressure.

Clearance of Cr-EDTA was used to measure skGFR. 3 MBq was injected as a bolus, followed by continuous infusion (2 MBq/h). The following hour was considered as a loading phase to stabilize the plasma concentration of Cr-EDTA. After this, urine was collected every 30 min for 2 h. A blood sample (4 ml) was taken every time a new collection of urine started, and by the end of the last urine collection. Blood samples were taken in a heparinized glass and centrifuged to isolate plasma. The mass of urine was estimated for each sample, and the volume was calculated using the assumption that the density of urine was 1 g/ml. Respective 2 ml of plasma and 0.1 ml of urine were diluted with 1.9 ml isotonic saline and analysed with a gamma counter (Packard Cobra II; Perkin-Elmer, Salem, MA). skGFR were calculated using the equation:

(1) skGFR = (Flow_urine_·C_urin_)/C_plasma_

### Stereological procedures

Nephrectomy was performed, and the kidney was perfusion-fixed through the renal artery. First, the kidney was flushed with 200 ml of phosphate buffer to drain blood from the kidney. Secondly, the kidney was flushed with phosphate-buffered formaldehyde (4%) using a peristaltic pump (250 ml/min) for approximately 5 min. The kidney was then immersion-fixed in phosphate-buffered formaldehyde (4%).

### Estimation of kidney volumes

Each kidney was transversally cut into slabs of 3 mm with a random initial cutting location, and every fifth slab was sampled with a random start to prevent bias (between 6 and 8 slices were sampled per kidney). A transparent point grid was put on top of each slab, and the number of points (P) hitting cortex/whole kidney was noted for all slabs. Cortex was defined macroscopically, and renal volumes (V(cor) and V(total)) were estimated by Cavalieri's principle using the following equation:

(2) V(cor or total) = Σ P(cor or total)·a(point)·t

where a(point) is the area pr. point and t is the slice thickness [9].

### N(glom) by physical fractionator

Kidney slabs were cut into blocks (1–16) from which one was sampled and embedded in plastic. Two slices, 30 μm thick, was cut from each block and put on glass slides and stained with Mayers haematoxylin. The slices made up a fraction of 1/41 of a block due to shrinkage during plastic embedding. For sampling of glomeruli we used the analysis software CAST (Visiopharm, Hørsholm, Denmark) and an Olympus BX50 microscope with a SIS-1 digital camera (Olympus Soft Imaging Solutions GmbH, Münster, Germany) and ×90 magnification. The microscope stage could be moved by a motor in predetermined, equidistant steps in two orthogonal directions, allowing the counting frame to cover a known fraction of the two slices, denoted the sampling section and the look-up section. Knowing the area of the counting frame, a(frame), and the length of the equidistant steps, dx and dy, the area sampling fraction (ASF) could be estimated as:

(3) $$ASF=\frac{a\left(frame\right)}{dx\cdot dy}$$

where a(frame) = 291·10^4 ^μm^2^, dx and dy = 4500 μm, resulting in ASF = 1/7. The total number of' glomeruli in one kidney was calculated as the sum of sampled glomeruli from all blocks multiplied with the inverse fractions:

(4) $$N{\left(glom\right)}_{kidney}=\frac{\sum Q_{block}^{-}}{2}\cdot {\left(\frac{1}{5}\cdot \left[\frac{1}{1};\frac{1}{16}\right]\cdot \frac{1}{41}\cdot \frac{1}{7}\right)}^{-1}$$

Where ∑Q^-^_block _is the number of glomeruli sampled from all blocks. Since glomeruli were sampled from both sample- and look-up section the area of the *actual *counting frame was 2·a(frame), and therefore ∑Q^-^_block _was divided with 2. The fraction 1/5 refers to the sampling of every fifth kidney slab, [1/1–1/16] to the sampling of kidney blocks (causing variable sampling fractions), 1/41 to the sampling of kidney slices and 1/7 to the sampling of area (using the counting frame).

### V(glom) by test point system, V(glom)^TPS^

We estimated the volume density of glomeruli, Vv(glom/cor), by point counting on 3 μm thick slices of renal cortex. Using the software CAST a test point system was displayed on a computer monitor together with live images of the slices, sampled by SURS. Test points hitting glomerular tuft, P(glom), and cortex (glomeruli included), P(cor), were sampled, and Vv(glom/cor) could be calculated as:

(5) $$\text{Vv(glom/cor)}=\frac{\sum \text{P}\left(\text{glom}\right)\cdot \text{p}\left(\text{cor}\right)}{\sum \text{P}\left(\text{cor}\right)\cdot \text{p}\left(\text{glom}\right)}$$

where p(glom) and p(cor) were number of test points per field-of-view used to sample glomeruli and cortex and were 30 and 48. The parameters dx and dy were each set to 5000 μm. From knowledge of the total volume of renal cortex, the total volume of glomeruli in renal cortex was calculated:

(6) *V*(*glom*) = *V*_*V*_(*glom*/*cor*)·*V*(*cor*)

### Statistics

As described, four successive time periods were used to measure skGFR. From these four periods, four separate estimates of skGFR were obtained. These estimates were analysed for outliers using Dixons test for extreme values, considering the tested value an outlier if the test quotient was above 0.765 (p < 0.05) [10]. After removing outliers, the skGFR period estimates were pooled to obtain an adjusted mean of skGFR.

To assure statistical independence between kidney groups in the case where both kidneys from one animal were used (UUOipsi and UUOcontra), the stereological parameters from the two kidney groups were compared by Pearson's correlation test. In no cases, this test indicated statistically acceptable correlation and therefore both kidneys were analysed. To evaluate methodological uncertainties, the coefficient of error (CE) was measured for each of the stereological parameters. The correlation between the stereological parameters and skGFR was analysed by Pearson's correlation test. The prediction of skGFR from stereological parameters was analysed by a linear regression test, from which graphs were performed showing the best prediction, as well as 95% safety and prediction intervals.

## Results

In the UUO group, two pigs were excluded due to congenital ureteral obstruction, and one pig died due to cardiac arrest during the experimental procedure. This resulted in 30 kidneys successfully used for functional and stereological measurements.

### skGFR measurements

The mass of urine for each sample was 9.7 – 154.4 g with a mean mass of 60.6 g and a median mass of 53.0 g. Five skGFR period estimates were found to be outliers and therefore excluded. The excluded values contained two periods estimates from the UUOipsi group and three from the UUOcontra group. Hereafter, the range of mean skGFR was 9.6 – 21.8 ml/min with a mean of 15.7 ml/min for UUOipsi, 18.9 – 30.2 ml/min with a mean of 24.9 ml/min for UUOcontra and 16.0 – 48.7 ml/min with a mean of 31.9 ml/min for the Controls.

### Correlation of stereological parameters to skGFR

Single kidney values concerning V(cor), V(total), N(glom), V(glom) and adjusted average of skGFR are shown in Table 1. The range was 36.2 – 85.1 cm^3 ^for V(cor), 57.2 – 115.6 cm^3 ^for V(total), 1.02·10^6^– 2.07·10^6 ^for N(glom) and 881 – 2039 mm^3 ^for V(glom).

**Table 1:** Stereological and skGFR values of kidneys.

**Kidney no**	**V(cor) (cm^3^)**	**V(total) (cm^3^)**	**N(glom) (×10^6^)**	**V(glom) (mm^3^)**	**skGFR (ml/min)**
**UUOipsi**					
#1	36.2	57.2	1.05	881	9.6
#2	41.1	77.8	1.10	996	15.1
#3	43.1	71.1	1.59	957	11.1
#4	42.9	61.9	1.11	1161	15.3
#5	59.3	80.8	1.75	1199	18.0
#6	58.9	88.7	1.32	1202	15.6
#7	49.7	71.4	1.02	1000	18.6
#8	46.9	75.0	1.17	1084	16.0
#9	58.3	81.4	1.62	1231	21.8
**Mean**	**48.5**	**73.9**	**1.30**	**1079**	**15.7**
					
**UUOcontra**					
#1	64.3	80.6	1.13	1241	19.8
#2	69.9	94.1	1.67	1632	30.2
#3	66.4	90.9	1.26	1315	18.9
#4	67.7	88.7	1.46	1527	27.9
#5	62.8	83.1	1.47	1454	21.4
#6	63.8	84.8	1.35	913	25.7
#7	64.7	87.8	1.24	979	25.5
#8	65.6	93.6	1.39	1514	30.1
#9	69.4	94.3	1.98	1452	24.7
**Mean**	**66.1**	**88.6**	**1.44**	**1336**	**24.9**
					
**Controls**					
#1	54.9	70.7	1.43	1621	16.0
#2	60.8	77.7	1.43	1889	29.2
#3	78.1	104.2	1.92	1953	48.7
#4	66.3	96.1	1.47	1995	25.5
#5	61.1	75.1	1.34	1237	35.8
#6	83.2	115.6	1.69	1590	34.7
#7	74.4	93.5	1.47	1418	32.2
#8	76.2	97.9	2.07	2039	47.1
#9	66.3	86.5	1.22	1726	32.1
#10	80.6	104.9	1.90	1601	39.6
#11	58.5	74.6	1.60	1261	36.3
#12	85.1	108.6	1.31	1528	29.7
**Mean**	**70.4**	**92.1**	**1.57**	**1655**	**33.9**

UUOipsi: relieved unilateral ureteral obstructed kidneys. UUOcontra: kidneys opposite to the UUOipsi. Controls: healthy kidneys.

**Figure 1:**
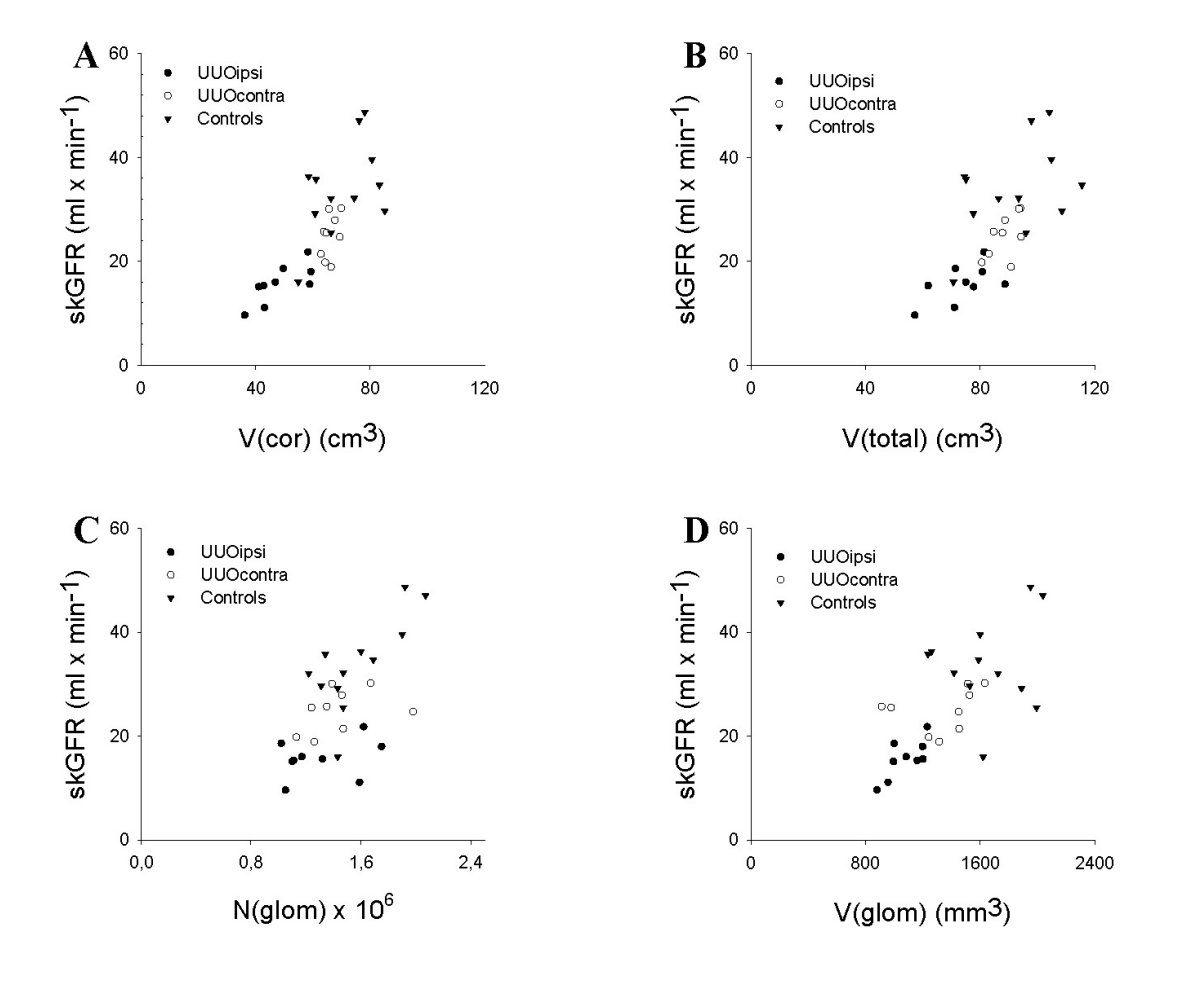
**Single-kidney glomerular filtration rate, skGFR, plotted as a function of the morphological parameters**. A: renal cortical volume, V(cor). B: total renal volume, V(total). C: number of glomeruli, N(glom). D: total volume of glomeruli, V(glom). Results are plotted for all pigs. UUOipsi: relieved unilateral ureteral obstructed kidneys. UUOcontra: kidneys opposite to the UUOipsi. Controls: healthy kidneys.

skGFR are plotted as a function of the stereological parameters in Figure 1. The results of the statistical correlations between the stereological parameters and skGFR are shown in Table 2, both stratified according to intervention, as well as pooled. For the UUO ipsilateral group V(cor) and V(glom) both showed significant positive correlations to skGFR, respectively (p < 0.05), whereas V(total) and N(glom) did not. In the UUO contralateral group none of the measured morphological parameters correlated significantly to skGFR, and in the control group N(glom) as the only parameter showed significant correlation. With the kidneys pooled, all of the measured morphological parameters correlated significantly to skGFR (p < 0.001), with r-values ranging from 0.62 for N(glom) to 0.78 for V(cor).

**Table 2:** Stereological parameters vs skGFR (Pearson's test).

	**r**	**p**
**UUOipsi**		
		
V(cor)	0.76	0.018
V(total)	0.58	0.099
N(glom)	0.31	0.425
V(glom)	0.74	0.024
		
**UUOcontra**		
		
V(cor)	0.46	0.217
V(total)	0.58	0.103
N(glom)	0.38	0.309
V(glom)	0.32	0.397
		
**Controls**		
		
V(cor)	0.50	0.098
V(total)	0.43	0.166
N(glom)	0.73	0.007
V(glom)	0.18	0.574
		
**All kidneys**		
		
V(cor)	0.78	<0.001
V(total)	0.66	<0.001
N(glom)	0.62	<0.001
V(glom)	0.68	<0.001

UUOipsi: relieved unilateral ureteral obstructed kidneys. UUOcontra: kidneys opposite to the UUOipsi. Controls: healthy kidneys.

### Prediction of skGFR using stereological parameters

**Figure 2:**
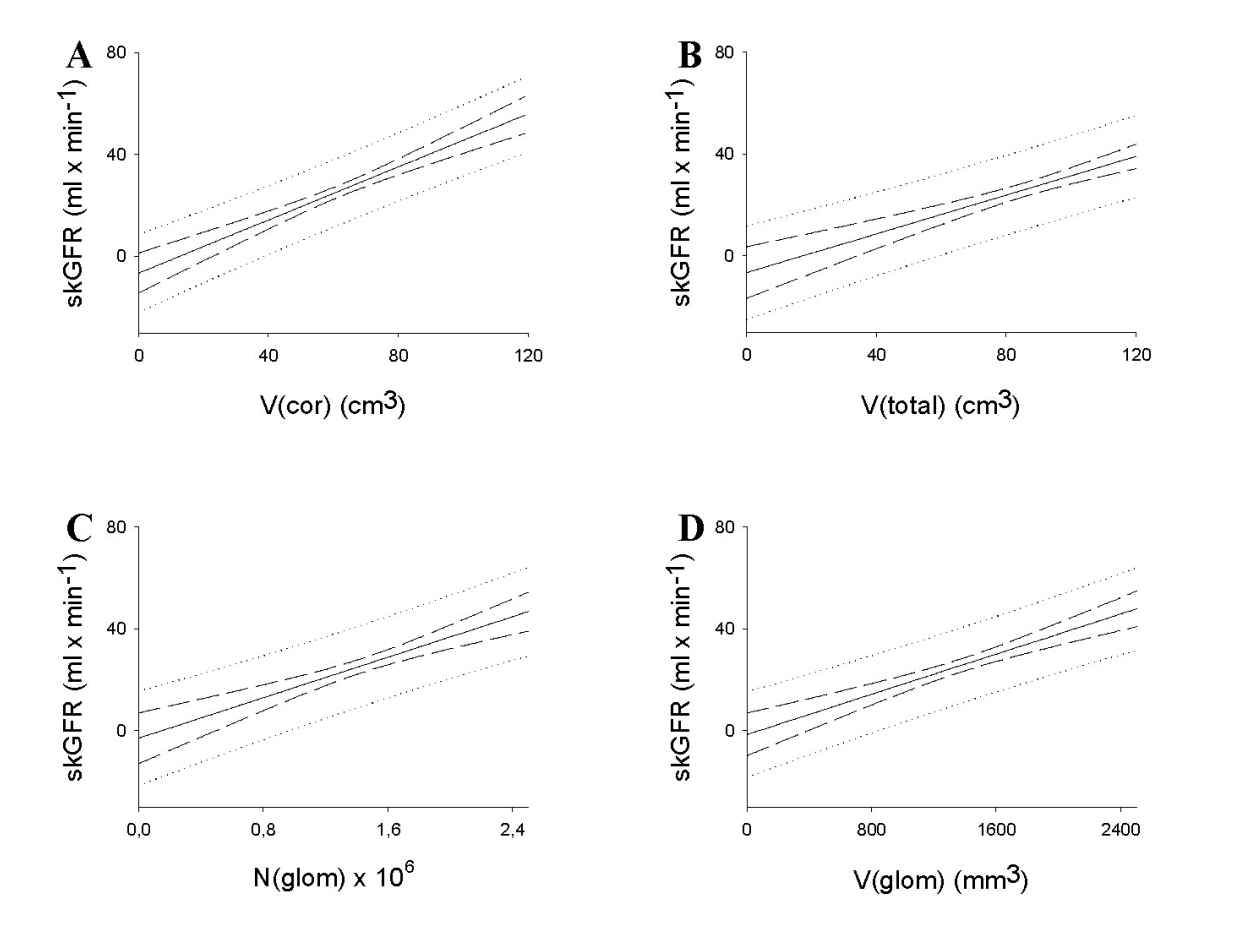
**Prediction of single-kidney glomerular filtration rate, skGFR, from the morphological parameters**. A: renal cortical volume, V(cor). B: total renal volume, V(total). C: number of glomeruli, N(glom). D: total volume of glomeruli, V(glom). The full-drawn line indicates the best prediction, the long dashed lines indicate the 95% confidence interval limits, and the dotted lines indicate the 95% prediction interval limits.

The prediction graphs are shown in Figure 2. The equations for the best prediction lines were as follows:

(7) skGFR = 0.618 ml/min/cm^3^·V(cor) - 12.9 ml/min

(8) skGFR = 0.487 ml/min/cm^3^·V(total) - 15.9 ml/min

(9) skGFR = 21.7 ml/min·10^-6^·N(glom) - 5.8 ml/min

(10) skGFR = 0.02 ml/min/cm^3^·V(cor) - 2.5 ml/min

### Precision of estimates

The total variation (CV(total)) for skGFR varied between 3% and 60% (data not shown). CV(total) of the stereological estimates were 25% and 18% for V(cor) and V(total), 19% for N(glom) and 13% for V(glom). CV(total) results from biological and methodological variance. The latter, expressed as the coefficient of error (CE), was 1% for V(cor) and V(total), and 9% for N(glom) and V(glom). Biological variance was 25% and 18% for V(cor) and V(total), 16% for N(glom) and 9% for V(glom). The ratio CE^2^/CV(total)^2 ^was used as a parameter of the impact CE had on CV(total). It was in all situations below 0.5 and therefore considered acceptable. Actual number of P sampled for estimation of V(total) and V(cor) were on average 231 and 166 pr. kidney, number of sampled glomeruli were 148 and number of sampled P(glom) and P(cor) were 277 and 807 pr. kidney (data not shown). For details concerning calculation of CE, please see [11].

## Discussion

Generally, we demonstrated a good correlation between structural parameters and skGFR. When all kidneys were evaluated together Pearson's correlation coefficient between skGFR and any stereological parameter was above 0.60 and highly significant (p < 0.001). The best correlation was found between V(cor) and skGFR. However, none of the structural parameters showed a significant correlation to skGFR in either group. A plausible explanation for this may be that the kidneys within a specific group varied relatively little with regards to structural parameters. Another explanation may be imprecision of skGFR period estimates (data not shown): five of the skGFR period estimates differed to such an extent they were classified as outliers. Different reasons may be responsible for this. The tubes used for urine collection contained approximately 3 ml of urine constantly, indicating that this amount of urine actually belonging to one period was left over for the next period. In addition, a quantity of urine might have been contained in the pelvis and upper ureter above the tube, due to reduced peristaltic activity in pelvis and ureter or transient clots in the tube, subsequently leading to a false low skGFR value in the first of these two periods, and a false high skGFR value in the second of the two periods. Furthermore, the differences found might derive from radioactive contamination of blood and urine samples leading to false low and false high GFR measurements, and imprecision with regards to measuring volumes and weight of urine and blood. Thus, double determinations of radioactive samples were performed in this study to avoid failures like these. Finally, some biologic variation of skGFR over time should be considered.

A study by D'Souza et al showed an excellent correlation between V(cor) and skGFR (r = 0.86) [12]. Widjaja et al found that renal length and V(total) both correlated to skGFR in patients with suspected renal artery stenosis, r = 0.75 for V(total) and r = 0.69 for renal length [2]. Considering our findings (r = 0.78 for V(cor) and r = 0.66 for V(total)) and the above mentioned study by D'Souza, it seems reasonable that V(cor) correlates better to skGFR than V(total) and renal length.

The capillary tuft has a rather complex three-dimensional configuration which may change with the size of the glomerulus. Also, the efficacy of the filtration membrane may differ accordingly. So which would give the highest skGFR, many small or a few big glomeruli, is not a question that can be answered intuitively. And this is also reflected in the correlations to skGFR. We found a trend towards V(glom) having the strongest correlation to skGFR when all pigs were evaluated as one group (0.68 for V(glom) and 0.62 for N(glom)). However, N(glom) was the only parameter which showed significant correlation to skGFR within the control group. Ureteral obstruction may have altered the filtration membrane, perhaps in both UUOipsi and UUOcontra, and this may have interrupted the straightforward relationship between N(glom) and skGFR. However, the imprecision of the skGFR period estimates reduces this to speculations.

Our findings are partly in accordance with findings reported by Fulladosa et al which found a correlation between N(glom) and GFR using biopsies from renal transplants (r = 0.42, P = 0.002) [13]. In a study by Almeida et al maternal gestational calorie-protein restriction was associated with a lower N(glom) and a decreased GFR in adult hypertensive rats compared to adult offspring from normally fed mothers, suggesting that the correlation shown in our study also corresponds to poorly developed kidneys [14].

All of the reported structural parameters were obtained in a design-based manner, which is the golden standard in stereology, as opposed to the way in which similar parameters are obtained in the clinic. The correlation between renal anatomy and function in humans may attract specific interest to the measurements of V(cor), because this parameter can be assessed non-invasively with high precision using MRI [15,16]. This is not (yet) possible for either N(glom) or V(glom), where kidney biopsies are needed [13,17]. However, the qualitative information obtained with a kidney biopsy is also an important supplement to the estimations of N(glom) or V(glom).

In this study, a UUO-model was used to imitate a chronic kidney disease and reduce the renal function. UUO-models have previously been used in other studies and are known to be associated with reductions in renal function [18,19]. The measured stereological parameters V(cor), V(total), N(glom), and V(glom), all indicated that UUO made an irreversible damage to the kidney (Table 1).

No comparisons of structural parameters and skGFR were made between pigs subjected to UUO and Controls since they were obtained from different litters.

As seen in Figure 2, the confidence intervals offered relatively narrow measurements, especially around the mean stereological values. The prediction intervals, however, presented wide measurements, indicating large uncertainties with regards to predicting skGFR from any stereological parameter, presumably due to imprecise skGFR measurements.

This experimental study confirms that a precise prediction of renal function from a single stereological parameter appears difficult to obtain. If another parameter that correlates well to renal function could be found independently from the stereological parameters, a multivariate prediction analysis hereby could be performed, and precise predictions would perhaps be possible.

## Conclusion

The correlations between the structural parameters and the kidney function in healthy as well as in chronically injured kidneys, suggest that these parameters in the future may be used as surrogate markers of kidney function. Even single kidney function may be estimated, an interesting feature considering the present use of radioisotope techniques for evaluating split kidney function. Which clinical compounds of kidney disease that may be monitored by these parameters is a challenge for future investigation. Furthermore, additional studies are needed to elucidate whether our findings can be extrapolated to human beings.

## Competing interests

The authors declare that they have no competing interests.

## Authors' contributions

ABL and KK participated in the design of the study, carried out the experiments, made the data analysis and statistical analysis and drafted the manuscript. THD participated in the design of the study, and assisted and supervised in the performance of the animal experiments as well as the data analysis. JRN participated in the design of the study, assisted and supervised in the stereological investigations, and contributed to the data interpretation and analysis as well as the statistical analysis. MP participated in the design of the study, performed the fundraising, assisted and supervised in the data interpretation and analysis, supervised the statistical analysis and manuscript writing.

All authors read and approved the final manuscript.

## Pre-publication history

The pre-publication history for this paper can be accessed here:


